# Maternal stress, child behavior and the promotive role of older siblings

**DOI:** 10.1186/s12889-022-13261-2

**Published:** 2022-04-29

**Authors:** Federica Amici, Stefan Röder, Wieland Kiess, Michael Borte, Ana C. Zenclussen, Anja Widdig, Gunda Herberth

**Affiliations:** 1 Department of Comparative Cultural Psychology, Max Planck Institute for Evolutionary Anthropology, Deutscher Platz 6, 04103 Leipzig, Germany; 2 Behavioural Ecology Research Group, Institute of Biology, University of Leipzig, 04103 Leipzig, Germany; 3 Department of Environmental Immunology, Helmholtz Centre for Environmental Research-UFZ, Leipzig, Germany; 4 Department of Women and Child Health, Center of Paediatric Research (CPL), Hospital for Children and Adolescents, Leipzig University, Leipzig, Germany; 5 Leipzig Research Centre for Civilization Diseases (LIFE), Leipzig University, Leipzig, Germany; 6 Children’s Hospital, Municipal Hospital “St. Georg”, Academic Teaching Hospital of the University of Leipzig, 04129 Leipzig, Germany; 7 German Centre for Integrative Biodiversity Research (iDiv) Halle-Jena-Leipzig, 04103 Leipzig, Germany; 8 Department of Human Behaviour, Ecology and Culture, Max Planck Institute for Evolutionary Anthropology, 04103 Leipzig, Germany

**Keywords:** Siblings, Risk factors, Promotive factors, Protective factors, Maternal stress, Child development

## Abstract

**Background:**

In the first years of their lives, children develop the cognitive, social and emotional skills that will provide the foundations for their lifelong health and achievements. To increase their life prospects and reduce the long-term effects of early aversive conditions, it is therefore crucial to understand the risk factors that negatively affect child development and the factors that are instead beneficial. In this study, we tested (i) the effects of different social and environmental stressors on maternal stress levels, (ii) the dynamic relationship between maternal stress and child behavior problems during development, and (iii) the potential promotive (i.e. main) or protective (i.e. buffering) effect of siblings on child behavior problems during development.

**Methods:**

We used longitudinal data from 373 mother–child pairs (188 daughters, 185 sons) from pregnancy until 10 years of age. We assessed maternal stress and child behavior problems (internalizing and externalizing) with validated questionnaires, and then used linear mixed models, generalized linear mixed models and longitudinal cross-lagged models to analyze the data.

**Results:**

Our results showed that higher maternal stress levels were predicted by socio-environmental stressors (i.e. the lack of sufficient social areas in the neighborhood). Moreover, prenatal maternal stress reliably predicted the occurrence of behavior problems during childhood. Finally, the presence of older siblings had a promotive function, by reducing the likelihood that children developed externalizing problems.

**Conclusions:**

Overall, our results confirm the negative effects that maternal stress during pregnancy may have on the offspring, and suggest an important main effect of older siblings in promoting a positive child development.

**Supplementary Information:**

The online version contains supplementary material available at 10.1186/s12889-022-13261-2.

## Introduction

In the first years of their lives, children develop the cognitive, social and emotional skills that will provide the foundations for their lifelong health and achievements [1]. In order to increase child life prospects and reduce the long-term effects of early aversive conditions, it is utterly necessary to understand both the risk factors that may negatively affect their healthy development and the factors that may instead promote or protect it [1–4]. According to the developmental origins of health and disease theory, exposure to environmental stressors during critical periods of life can have negative long-term consequences for children’s health and development [5–7]. Parental stress (i.e. maternal or paternal stress), for instance, which is caused by a variety of social and environmental factors, can have serious short- and long-term effects on children [8, 9], by increasing their risk of developing diseases and behavior problems (e.g. [10–12]). Families, however, can have a promotive (i.e. main) and protective (i.e. buffering) function against the negative effects of stress on child development. Positive sibling relationships, for instance, may reduce the occurrence of behavior problems in children [13, 14], or mitigate the negative consequences of stressful events that children experience [15, 16]. In this study, we therefore explored (i) the effects of different socio-environmental stressors on maternal stress levels, (ii) the dynamic relationship between maternal stress (including prenatal stress) and child behavior problems during development, and (iii) the potential promotive or protective function of siblings toward the emergence of child behavior problems.

### Socio-environmental factors contributing to maternal stress

Among the most studied risk factors for child health and development are those linked to parental stress [8, 9]. Depending on the characteristics of the stressors (e.g. their intensity, duration or predictability), and on the individual life histories (e.g. early exposure to stress [17, 18]), stressors can trigger adaptive and/or maladaptive responses, allowing individuals to optimally respond to potential threats and/or triggering the disruption of stress coping mechanisms, respectively [19]. When stressors are too intense, or too long, individuals may not manage to cope with them, i.e. the stress response system may remain activated and there can be important negative consequences on individual health [20].

Several stressors can activate the parental stress response system [21–26], including acute disasters (e.g. terroristic attacks or droughts), psychological and physiological changes linked to maternity, work-related stress and even physical and emotional challenges experienced during everyday life [1, 21, 22, 27]. Parents, for instance, show higher levels of stress when they receive little social support, when their offspring experiences health or behavior problems, in case of conflict among family members, or in the presence of enduring socio-environmental stressors, like deprived environments [23–26]. Individuals who are satisfied with the environment they live in, in contrast, may experience lower stress levels: a safe neighborhood characterized by high environmental quality (e.g. in terms of air quality), the presence of social areas, meeting places, shops and other infrastructures, for example, may all contribute to increase neighborhood satisfaction and well-being [28–30] and hence decrease individual stress levels [31]. Similarly, individual stress levels may be lower when the quality of social relationships with neighbors is high, as neighbors can contribute to social integration and individual well-being (e.g. [32, 33]).

### Prenatal stress as a long-term risk factor for child development

When parents are stressed, children have a higher risk of developing social, emotional, behavior and cognitive problems [8, 9, 34–36]. Maternal stress can have a negative impact on offspring development already in the utero [37–39], and even before conception [40]. Prenatal stress may have adverse effects on fetal development through an increase of stress hormones during pregnancy [41, 42], placental inflammation [43, 44], or by altering epigenetic regulation through changes in mRNA expression and DNA methylation [45, 46]. The consequences for children can be substantial. Prenatal maternal stress can modify the fetal immune system [47], affect birth outcome [48, 49] and lead to several diseases during childhood, like obesity or wheezing [50, 51].

Prenatal stress can also have negative effects on offspring psychosocial development [52–54], as well as brain and cognitive development [52, 55–57]. Moreover, stress during pregnancy might increase the likelihood of behavior problems during childhood [54, 58], including attention deficit or hyperactivity [53, 59], conduct disorders [53, 60], and even increase the occurrence of autism [61], depression and schizophrenia in children [62]. Prenatal stress, for instance, reliably predicts the occurrence of internalizing (e.g. anxiety, poor self-estimation) and/or externalizing (e.g. hyperactivity, aggression) behavior problems in children (e.g. [12, 58, 63–68]), even when accounting for maternal stress levels after pregnancy (e.g. [69, 70]. These findings are not restricted to extreme prenatal stress or response to acute disasters (e.g. terroristic attacks or droughts), but they may also extend to maternal stress caused by daily challenges, pregnancy anxiety or relationship strain [21, 22].

### The link between maternal stress and child behavior problems

Maternal stress can also be linked to the occurrence of offspring behavior problems in the short term. Several studies have shown a clear link between the occurrence of parental stress and children’s internalizing and externalizing behavior problems (e.g. [71–75]). When mothers suffer from stress, for instance, children consistently show an increasing risk of developing internalizing problems, like depression and anxiety, and externalizing problems, like aggressive and rule-breaking behavior [75].

The relationship between maternal stress and child behavior problems, however, is complex, as parents and children reciprocally affect each other. Whereas children are more likely to develop behavior, social or emotional problems if their parents suffer from stress, children with these problems may in turn increase parental stress, giving rise to a cycle of negative parent–child interactions with adverse outcomes for all parties involved [8, 34, 76–79]. Longitudinal studies and methodological approaches (e.g. cross-lagged models) that assess how parental stress and child behavior problems reciprocally affect each other are therefore necessary to disentangle to what extent maternal stress contributes to child behavior problems, and/or the other way round, and/or whether other external factors can explain the link between these two variables (see e.g. [8]).

### The role of siblings as protective or promotive factor

Among the most important environmental factors that affect child development are parental and family well-being (e.g., [80–82]). On the one hand, the family environment can entail risk factors for the child development, including parental stress (see above). On the other hand, families have an essential promotive or protective function for children’s healthy development, in the face of aversive conditions. Although there is no consensus in literature, promotive factors are usually considered those factors that have direct ameliorative effects on child development, being linked to a general decrease in behavior problems (i.e. as main effect), whereas protective factors usually indicate those factors that buffer children from the occurrence of behavior problems in the face of risk (i.e. in interaction with other factors; [4]). A healthy social environment, for instance, can provide increased social support to parents, directly reducing their stress levels and increasing their psychological well-being (i.e. promotive function), or it can have an indirect function by mitigating the negative consequences of parental stress on children (i.e. protective function) [83, 84].

To date, several studies have shown that siblings can significantly contribute to family well-being. Siblings are an essential part of family systems [85], they usually have long-lasting relationships with each other and spend abundant time together [86–88]. Therefore, siblings may learn to understand each other’s thoughts, emotions and intentions from early on, facilitating the development of social, emotional and cognitive skills [89–91]. Moreover, siblings may serve an important promotive or protective function during child development, reducing the occurrence of child behavior problems and/or mitigating the negative consequences of stressful events [13–16]. By generally contributing to a healthy social and cognitive development, positive sibling relationships may indeed reduce the likelihood that children show adjustment problems (e.g. [87]; for reviews, see [13, 14]). Children with siblings, for instance, are less likely to develop internalizing problems, even when controlling for maternal stress levels [75].

Moreover, siblings may exert a protective function for children, by reducing the risk that adverse conditions trigger the emergence of internalizing and externalizing problems [15, 16]. Positive sibling relationships, for example, can decrease the probability that children develop internalizing problems after experiencing stressful events [15]. However, the mechanisms beyond these processes are not clear. Siblings, especially older ones, may provide each other comfort and security, or they may more successfully distract each other when experiencing stress [15]. Either way, siblings may have a protective function in case of adverse conditions, increasing their ability to positively react to environmental stressors [15, 92].

Although longitudinal studies are especially important to further explore the promotive and protective function of siblings and temporally disentangle the complex interplay of parental stress and child behavior problems, such an approach has rarely been used [15]. However, it can be crucial to understand how parental stress and child behavior problems are linked to each other through time, and whether the presence of siblings provides direct or indirect benefits to children during their development. Moreover, siblings can have a different role depending on their age and gender. While older siblings are usually more prone to engage in caregiving and helping roles (and their protective/promotive function may thus be especially relevant), younger siblings are usually more likely to elicit help and care (see [91]). Furthermore, some studies have shown that older sisters are more likely to engage in caretaking and helping behavior toward siblings, as compared to older brothers [93, 94]. Therefore, separately assessing the role of older brother and sisters may be essential to understand the protective and/or promotive function that siblings may have during child development.

### The present study

In this study, we used longitudinal data on maternal stress levels and child behavior to test (i) the effects of different social and environmental stressors on maternal stress levels, (ii) the dynamic relationship between maternal stress and child behavior problems during development, and (iii) the potential promotive/protective function of siblings during child development. In this way, we aimed to contribute novel findings on the complex interplay of socio-environmental stressors, maternal stress and child behavior problems, by especially focusing on the role of promotive/protective factors in this relationship. Moreover, we aimed to confirm previous findings linking maternal stress and child behavior problems—an important endeavor in psychology to increase reproducibility (e.g. [95]). Based on previous studies (see above), we made the following predictions. First, we predicted that maternal stress (which we assessed when children were aged 10) would be higher when mothers are less satisfied with the environment they live in, in terms of living standards (e.g. air quality, safety) and quality of social relationships with neighbors. Second, we predicted that higher levels of maternal stress would be linked to an increased probability of internalizing (e.g. anxiety, poor self-estimation) and/or externalizing (e.g. hyperactivity, aggression) behavior problems in children. In particular, we predicted that prenatal maternal stress would have a negative long-term effect on offspring behavior during childhood (which we assessed when children were 7, 8 and 10), whereas maternal stress and behavior problems (both assessed when children were 7, 8 and 10) would be linked to each other throughout development. As maternal stress and behavior problems during childhood may reciprocally affect each other, we further used longitudinal cross-lagged models to better disentangle the link between these variables. Finally, we predicted that the presence of older siblings would exert a protective or promotive function for children [15, 16], by buffering the negative effects of maternal stress on child behavior or by overall reducing the occurrence of behavior problems in children through their development (i.e. when children were 7, 8 and 10).

## Methods

### Participants

**Fig. 1:**
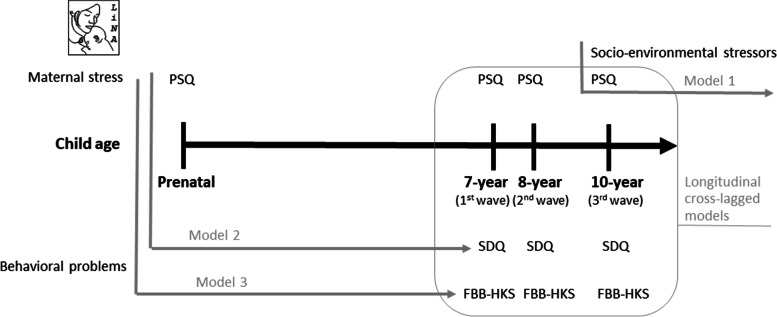
Pictorial representation of the set-up, summarizing in black the moments in which data on maternal stress (PSQ questionnaire) and child behavior problems (SDQ and FBB-HKS questionnaires) were collected, and in grey the statistical analyses run (with arrows indicating generalized linear mixed models and linear mixed models, and the square indicating the longitudinal cross-lagged models). Generalized linear mixed models were used to assess the factors predicting maternal stress levels (Model 1), the link between maternal stress (i.e. prenatal and postnatal) and child behavior problems, and the potential promotive or protective effect of siblings on child behavior problems (Models 2 and 3). Longitudinal cross-lagged models were used to study the dynamic relationship between maternal stress and behavior problems during child development (i.e. in the three waves)

This study was based on the prospective mother–child cohort LINA (Lifestyle and environmental factors and their Influence on Newborns Allergy risk) and included longitudinal data on maternal stress levels and child behavior from pregnancy until the age of 10 years of children life. We initially screened the data for 629 mother–child pairs recruited between March 2006 and December 2008 in Leipzig, Germany, among Caucasian mothers who did not suffer from severe infectious or immune diseases during pregnancy [96]. In the analyses presented here, we only included a LINA sub-cohort of 373 mother–child pairs for which information was available on both child behavior and maternal stress levels (see below; Fig. 1). For an overview of the main characteristics of the LINA sub-cohort analyzed in this study, including measures of socio-economic status, please refer to Table S1 in Suppl. Material. Participants were assessed longitudinally with standardized questionnaires (see below) self-administered by the parents, which measured (i) maternal stress levels when the mother was pregnant (i.e. 34^th^-36^th^ week of gestation), and (ii) both maternal stress and child behavior in three waves (i.e. when the child was 7, 8 and 10 years of age; Fig. 1). All mothers signed an informed consent.

### Maternal stress

We assessed maternal stress using a 20-item perceived stress questionnaire (PSQ [97, 98]). The PSQ is a validated questionnaire commonly used to assess how often (on a 4-point scale) mothers experience stress (e.g. worries, loss of joy, tension). For each questionnaire, we summed all the scored answers to each item (from 0 = almost never to 3 = mostly) and then divided the sum for the maximum score that could be obtained (i.e., 60), in order to obtain individual indices of maternal stress ranging from 0 to 1 (i.e., from less to more stressed; see [98]). Maternal stress was assessed longitudinally, first during pregnancy, and then in the three waves (i.e. when the child was 7, 8 and 10 years of age; Fig. 1). Internal consistency for this questionnaire was high, with Cronbach’s alpha ranging from 0.92 to 0.94 in the three waves.

### Child behavior: internalizing and externalizing behavior problems

Mothers were asked to assess their children’s behavior in the previous six months, at the three waves (i.e. when the child was 7, 8 and 10 years of age), using the standardized 25-item Strength and Difficulties Questionnaire (SDQ) that assesses internalizing and externalizing problems, including emotional, mental and behavioral ones [99]. More specifically, the SDQ questionnaire contained five questions for each of the following five dimensions: (i) hyperactivity/lack of attention (e.g. how easily the child is distracted), (ii) emotional symptoms (e.g. how often the child is unhappy or tearful), (iii) conduct problems (e.g. how often the child has temper tantrums), (iv) peer relationship problems (i.e. how solitary the child is), and (v) prosocial behaviors (i.e. how helpful the child is when others are hurt or upset). Given that only the first four dimensions refer to behavior problems [99], we first aggregated the four dimensions by summing the answers to the respective items that were assessed using a response format from 0 (not true) to 2 (true), obtaining a total difficulties score that ranged between 0 and 40. For modelling purposes (i.e. to model dependent variables as proportions, varying from 0 to 1), we then divided this score by the maximum score that could be obtained (i.e., 40; see [99]), so that the individual indices ranged from 0 to 1. Internal consistency for this questionnaire was only moderate (with Cronbach’s alpha ranging from 0.53 to 0.62 in the three waves), as expected given that the questionnaire includes different dimensions [99].

### Child behavior: externalizing behavior problems

We further asked mothers to assess child behavior in the three waves (i.e. when the child was 7, 8 and 10 years of age), using an adapted version of the Fremdbeurteilungsbogen für hyperkinetische Störungen (FBB-HKS), which provides a 20-item external assessment of child hyperkinetic disorders [100], and thus a more specific focus on children’s externalizing problems. Mothers were provided with 18 questions (see supplementary material for the list of questions) to be assessed on a 4-point scale (e.g. how often the child seems not to be listening when being talked to). As above, we calculated a second behavioral index ranging from 0 to 1 (i.e. from less to more problematic), by summing the scored answers (from 0 to 3, with 3 always indicating higher behavior problems) and then dividing the sum for the maximum score that could be obtained (i.e. 54). Internal consistency for this questionnaire was high, with Cronbach’s alpha ranging from 0.90 to 0.91 in the three waves. Behavior problems as assessed with the SDQ and the FBB-HKS questionnaires correlated in the three waves (Spearman exact test: *p* < 0.001, *N* = 251, rho in the first wave = 0.677, in the second wave: 0.746, and in the third wave: 0.742).

### Other information

In the three waves (i.e. when children were aged 7, 8 and 10), mothers were also asked for information on the number of siblings at home, their sex and age, and their own education level (on a 7-point scale, from pre-primary education to second stage of tertiary education; see [101]). In the third wave (i.e. when children were 10), we also used subjective measures to assess the potential impact of socio-environmental stressors, by asking mothers how satisfied they were with the environment they lived in, using 16 questions on a 4-point scale (i.e. with 0 meaning low satisfaction, and 3 high satisfaction). We included questions on mothers’ evaluation of their living standards, including (i) natural environment (e.g. air quality), (ii) safety of the area, (iii) presence of social areas and meeting places (e.g. playgrounds), (iv) shops and (v) other infrastructures (e.g. public transport). We thus calculated 5 indexes ranging from 0 to 1 (i.e. from lower to higher satisfaction), by summing the relative scored answers and dividing the sum for the maximum score that could be obtained. We further included 5 questions on the quality of social relationships with the neighbors, 2 on a 4-point scale (e.g. how good is the relationship to the neighbors) and 3 as polar questions (e.g. whether there are friends in the area, who are regularly met). As above, we calculated an index from 0 to 1 (i.e. from lower to higher satisfaction), by summing the scored answers and dividing the sum for the maximum score that could be obtained (i.e. 9). Therefore, these indexes were higher when mothers rated themselves as happier with their environment, and lower when they were less satisfied with it. Finally, mothers in the third wave (i.e. when children were 10) also provided information on the monthly family income (as a categorical variable from 0 to 9, with 0 being a lower income and 9 a higher one).

### Statistical analyses

We used linear mixed models, generalized linear mixed models and longitudinal cross-lagged models to analyze the data. For this purpose, we prepared three data-sets, (i) to study the factors predicting maternal stress levels (Model 1); (ii) to assess the link between maternal stress (i.e. prenatal and postnatal) and child behavior problems, and the potential promotive or protective effect of siblings on child behavior problems (Models 2 and 3); and (iii) to study the dynamic relationship between maternal stress and behavior problems during child development (i.e. in the three waves; longitudinal cross-lagged models). In the first data-set we entered one line for each child (i.e. only including the third wave; *N* = 268; Fig. 1). We then entered the individual indices for maternal stress levels in the third wave, maternal satisfaction (i.e. with neighbors, natural environment, safety of the area, presence of social areas, shops and other infrastructures), family income (as low income has been associated to increased stress, e.g. [102]), maternal education level (as education may have a protective function against stress, e.g. [103]) and number of children in the household (as the presence of more children at home can increase parental stress levels, e.g. [104]). In the second data-set, we entered one line for each child and wave (i.e. up to three lines per child, as tested at 7, 8 and 10 years of age; Fig. 1). As some children were not tested at all ages, we ended up with *N* = 974 lines in this data-set. We then entered child identity, SDQ and FBB-HKS behavioral indexes, maternal stress as assessed (i) prenatally and (ii) in the three waves, the number of siblings living in the same household (i.e. the overall number, the number of older sisters and the number of older brothers), the child’s gender (because males were more likely be reported to show behavioral problems, e.g. [105]), the child’s age (because the occurrence of behavioral problems may vary through development), and maternal education level (as this may have a positive impact on child development, e.g. [106, 107]). Further controls (e.g. family income) could not be added in this data-set, as this information was not available for all waves. In the third data-set, we entered one line for each child (*N* = 373), including maternal stress levels and SDQ and FBB-HKS behavioral indexes for each of the three waves, and further specifying whether the child had older siblings. We never aggregated data of the three waves.

Data were analyzed using R (version 3.5.0 [108]). We first used the glmmTMB package (version 1.0.1 [109]) to build three linear mixed models and generalized linear mixed models (Models 1 to 3; Fig. 1). In order to test for the role of social and environmental factors on maternal stress levels, we run the first linear mixed model with the first data-set, using maternal stress in the third wave as the dependent variable (Model 1). As predictors, we included several measures of maternal satisfaction with the environment (i.e. relationship quality with neighbors, natural environment, safety of the area, presence of social areas, shops and other infrastructures). We further controlled for the number of children in the household and for family income (which is known to reliably predict parental stress [110]).

In order to test for the link between maternal stress and child behavior problems, as well as the potential protective effect of siblings, we ran two generalized linear mixed models with the second data-set, using the SDQ (Model 2) and FBB-HKS (Model 3) behavioral indexes in the three waves, respectively, as dependent variables (Fig. 1). In both models, we included prenatal maternal stress and maternal stress in the wave as test predictors. To assess the protective role of siblings as social buffers against maternal stress, we further included as predictor the three 2-way interactions of maternal stress in the three waves with i) the overall number of siblings at home, ii) the number of older brothers and iii) the number of older sisters, respectively, and their main terms. If the 2-way interactions were non-significant, the interaction was removed and the model was re-run by including only the single predictors, thus allowing us to test for the promotive role of siblings on child behavior problems. We also included children’s sex as predictor, as sex may also predict behavior problems in children [111]. Finally, we controlled for children’s age (as our data were collected in three different waves), and included child identity as random factor to control for lack of independence, as the same children were entered multiple times in the data-set.

As the dependent variables in Models 1 to 3 were modelled as proportions including 0 and 1, we used a beta distribution after transforming them [112]. We also *z*-transformed continuous test predictors (i.e. age) to facilitate convergence. We compared full models to null models (i.e. only containing control predictors and random factors) using likelihood ratio tests [113]. When full and null models significantly differed, we obtained the *p* values for each test predictor using the R function summary. We detected no over-dispersion, convergence or stability issues in our models. There was also no problem of collinearity in our models (maximum VIFs across all models = 2.08).

In order to test for the dynamic relationship between changes in maternal stress and changes in child behavior problems during development, we run a further set of models, using the third dataset and the lavaan package (version 4.0 [114]) to build longitudinal cross-lagged models [115] (Fig. 1). These longitudinal models allow to disentangle the interplay of two variables across waves, and thus better infer possible causal pathways linking the two variables. In particular, it is possible to assess both cross domain-relationships (i.e. the predictive effect of one variable on the other one as tested in the following wave) and autocorrelations (i.e. the predictive effect of one variable on the same variable as tested in the following wave). In these longitudinal models, we always used full information maximum likelihood for missing data to reduce bias and increase statistical power [115]. We compared models of increasing complexity (see below) using likelihood ratio tests and also reported fit indices for the individual models, including robust Root Mean Square Error of Approximation (RMSEA), robust Comparative Fit Index (CFI), robust Tucker-Lewis Index (TLI) and Standardized Root Mean Square Residual (SRMR).

To run our longitudinal cross-lagged models, we first constructed child behavior problems as a latent variable, measured by a set of two observed measurements (i.e. SDQ and FBB-HKS behavioral indexes). We established measurement invariance through time by using a multiple indicator univariate model. In particular, we established configural, metric and scalar invariance by sequentially constraining factor loadings, error terms and intercepts through time [115]. All models fit the data well (configural model with no parameter constraints: χ^2^ = 19.28, *df* = 3, RMSEA = 0.130, CFI = 0.985, TLI = 0.925, SRMR = 0.024; metric model with factor loading and error term invariance: χ^2^ = 20.09, *df* = 11, RMSEA = 0.066, CFI = 0.986, TLI = 0.980, SRMR = 0.037; scalar model with intercept invariance: χ^2^ = 24.00, *df* = 13, RMSEA = 0.064, CFI = 0.984, TLI = 0.982, SRMR = 0.038). Crucially, there were no significant differences between models with increasing constraints (configural-metric model comparison: χ^2^ = 7.61, *df* = 8, *p* = 0.473; metric-scalar model comparison: χ^2^ = 4.23, *df* = 2, *p* = 0.121), suggesting scalar measurement invariance for child behavior problems through time.

After establishing measurement invariance, we tested for possible relationships between changes of maternal stress and changes in child behavior problems during the three waves (i.e. at 7, 8 and 10 years of age), using a longitudinal cross-lagged model. We decomposed each of the two variables into its score in the preceding wave and its change from the preceding to the current wave [115]. We then tested all possible relationships between maternal stress and child behavior problems: (i) stress-behavior covariance in the first wave, (ii) stress to behavior coupling, to assess whether stress scores in one wave predicted rates of changes in behavioral scores, (iii) behavior to stress coupling, to test whether behavioral scores in one wave predicted rates of changes in stress scores, and (iv) estimates of correlated changes, to assess whether stress-behavior changes co-occur after accounting for the coupling pathways (see [115] for details). In the model, we also specified an auto-regression parameter for maternal stress and another one for child behavior problems, to measure how change in each variable depended on the scores in the preceding wave. To obtain finer-graded measures of these relationships, and given the low number of waves in our model, we only assessed changes between adjacent waves [116]. Finally, we tested whether the relationships between maternal stress and child behavior problems differed for children with or without older siblings, and assessed whether this more complex model provided an increase of fit as compared to the preceding one [115].

## Results

**Fig. 2:**
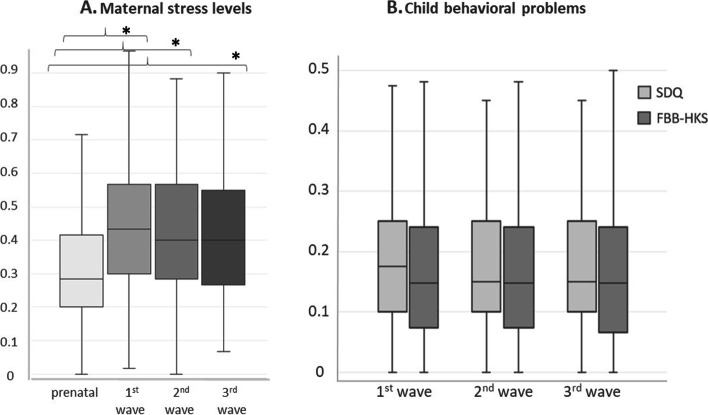
Boxplots representing data distribution for **A** maternal stress levels, as assessed with the PSQ questionnaire during pregnancy and in the three waves (i.e. when the child was 7, 8 and 10 years old); and for **B** child behavior problems, as assessed with the Strength and Difficulties Questionnaire (SDQ) and the Fremdbeurteilungsbogen für hyperkinetische Störungen questionnaire (FBB-HKS), in the three waves. The horizontal ends of the boxes represent the upper and lower quartiles, and the ends of the whiskers represent the maximum and minimum indices, excluding outliers. Asterisks denote significant differences

With regards to their behavior problems as scored with the SDQ, children in our study were in the 45^th^ percentile at age 7, and in the 44^th^ percentile at age 8 and 10, as compared to normative data for the German population [117, 118]. In particular, whereas they scored above the 50^th^ percentile for emotional symptoms in all waves (54^th^-59^th^), they always scored below the 50^th^ percentile for all the other dimensions (hyperactivity/lack of attention: 42^nd^-47^th^; conduct problems: 42^nd^-43th; peer relationship problems: 36^th^-40^th^). Moreover, most mothers (69% on average) reported a *low* incidence of behavior problems in their children (i.e. SDQ indices between 0 and 0.25; at 7 years: 70%, at 8 years: 69%, at 10 years: 68%). Further 26% of mothers reported an *intermediate* incidence of behavior problems (i.e. 0.25 ≤ SDQ indices < 0.50; 26% across all age classes). The minority of mothers reported either *no* child behavior problems (at 7 years: 2%; at 8 years: 3%; at 10 years: 3%), or *frequent* problems (i.e. SDQ ≥ 0.50; at 7 years: 2%; at 8 years: 2%; at 10 years: 4%). Figure 2 summarizes the mean indices for maternal stress levels (as assessed with the PSQ questionnaire) and for child behavior problems (as assessed with the SDQ and FBB-HKS questionnaires) in the three waves. Perceived maternal stress increased across child development. On average (± SD), mothers reported a PSQ index of 0.31 (± 0.16) during pregnancy, which increased to 0.43 (± 0.19) in the first wave (i.e. when the child was 7), 0.41 (± 0.19) in the second wave (i.e. when the child was 8) and 0.42 (± 0.19) in the third wave (i.e. when the child was 10). Therefore, maternal stress was significantly higher in the first, second and third wave, as compared to prenatal stress levels (exact Wilcoxon test for prenatal vs. the first wave: *N* = 235, *z* = -9.261, *p* < 0.001; vs. the second wave: *N* = 227, *z* = -7.879, *p* < 0.001; vs. the third wave: *N* = 229, *z* = -8.620, *p* < 0.001; Fig. 2).

In Model 1, we analyzed the effects of different social and environmental stressors on maternal stress levels. We detected a significant difference between the full and the null model (*χ*^2^ = 22.14, df = 6, *p* = 0.001; Table 1). In particular, the probability of reported maternal stress was higher when mothers were not satisfied with the presence of social areas in the place they lived (*p* = 0.017, *est* = -0.72, *se* = 0.30, *z* = -2.39; Fig. S1), while no other test predictor had a significant effect in our study.

**Table 1:** For each model, estimates, standard errors (SE), confidence intervals (CIs), *z* and *p* values of test and control predictors

MODEL	Estimate	SE	2.5% CI	97.5% CI	*z* values	*P*
**M1: Probability of reporting maternal stress in the PSQ questionnaire in the 3**^**rd**^** wave**						
Intercept	1.00	0.34	0.33	1.66	2.92	0.004
Relationship with neighbors	-0.48	0.28	-1.02	0.07	-1.70	0.090
Natural environment	0.12	0.34	-0.54	0.78	0.36	0.722
Safety of the area	-0.16	0.33	-0.80	0.48	-0.48	0.630
Availability of social areas	-0.72	0.30	-1.31	-0.13	-2.39	0.017*
Availability of shops	-0.15	0.25	-0.65	0.34	-0.61	0.542
Availability of other infrastructures	-0.41	0.37	-1.13	0.32	-1.10	0.272
*Maternal education*	0.02	0.05	-0.08	0.12	0.36	0.717
*Family monthly income*	-0.03	0.02	-0.08	0.01	-1.36	0.174
*Number of children in household*	-0.05	0.06	-0.18	0.07	-0.87	0.382
**M2: Probability of reporting child behavior problems in the SDQ questionnaire in the 3 waves**						
Intercept	-1.97	0.19	-2.35	-1.59	-10.12	< 0.001
Child gender (ref. category: female)	0.26	0.07	0.12	0.41	3.51	< 0.001 *
Maternal stress (prenatal)	0.78	0.26	0.27	1.28	3.03	0.002 *
Maternal stress (in the 3 waves)	0.72	0.18	0.38	1.07	4.09	< 0.001 *
Number of siblings	0.02	0.05	-0.07	0.11	0.35	0.728
Number of older sisters	-0.10	0.10	-0.29	0.09	-0.99	0.324
Number of older brothers	-0.12	0.10	-0.31	0.07	-1.25	0.211
*Maternal education*	-0.07	0.04	-0.15	0.00	-1.87	0.061
*Child age*	-0.01	0.02	-0.05	0.02	-0.73	0.464
**M3: Probability of reporting child behavior problems in the FBB-HKS questionnaire in the 3 waves**						
Intercept	-2.42	0.25	-2.92	-1.92	-9.52	< 0.001
Child gender (ref. category: female)	0.47	0.10	0.28	0.67	4.84	< 0.001*
Maternal stress (prenatal)	0.82	0.33	0.16	1.47	2.45	0.014*
Maternal stress (in the 3 waves)	0.82	0.21	0.42	1.23	3.98	< 0.001*
Number of siblings	0.10	0.05	-0.01	0.20	1.79	0.073
Number of older sisters	-0.24	0.12	-0.47	-0.01	-2.01	0.044*
Number of older brothers	-0.42	0.12	-0.65	-0.19	-3.60	< 0.001*
*Maternal education*	-0.05	0.05	-0.15	0.06	-0.91	0.365
*Child age*	-0.01	0.02	-0.05	0.02	-0.71	0.477

In Models 2 and 3, we tested the link between maternal stress and child behavior problems, and the potential protective effect of older siblings. For Model 2, we found a significant difference between the full and the null model (*χ*^2^ = 58.33, df = 9, *p* < 0.001; Table 1). In particular, the probability of reporting child behavior problems in the SDQ questionnaire was higher for male children (*p* < 0.001, *est* = 0.26, *se* = 0.07, *z* = 3.51), when mothers reported higher prenatal stress (*p* = 0.002, *est* = 0.78, *se* = 0.26, *z* = 3.03; Fig. S2a) and when they were more stressed in that wave (*p* < 0.001, *est* = 0.72, *se* = 0.18, *z* = 4.09; Fig. S2b).

The full and the null model also significantly differed for Model 3 (*χ*^2^ = 74.17, df = 9, *p* < 0.001; Table 1). The probability of reporting child behavior problems in the FBB-HKS questionnaire was higher for male children (*p* < 0.001, *est* = 0.47, *se* = 0.10, *z* = 4.84), when mothers reported higher prenatal stress (*p* = 0.014, *est* = 0.82, *se* = 0.33, *z* = 2.45; Fig. S2c) and when mothers reported higher stress in that wave (*p* < 0.001, *est* = 0.82, *se* = 0.21, *z* = 3.98; Fig. S2d). In addition, the presence of older brothers (*p* < 0.001, *est* = -0.42, *se* = 0.12, *z* = -3.60) and older sisters (*p* = 0.044, *est* = -0.24, *se* = 0.12, *z* = -2.01) was linked to a lower probability of reporting child behavior problems, regardless of maternal stress levels, suggesting a promotive (i.e. main) effect of older siblings.

**Fig. 3:**
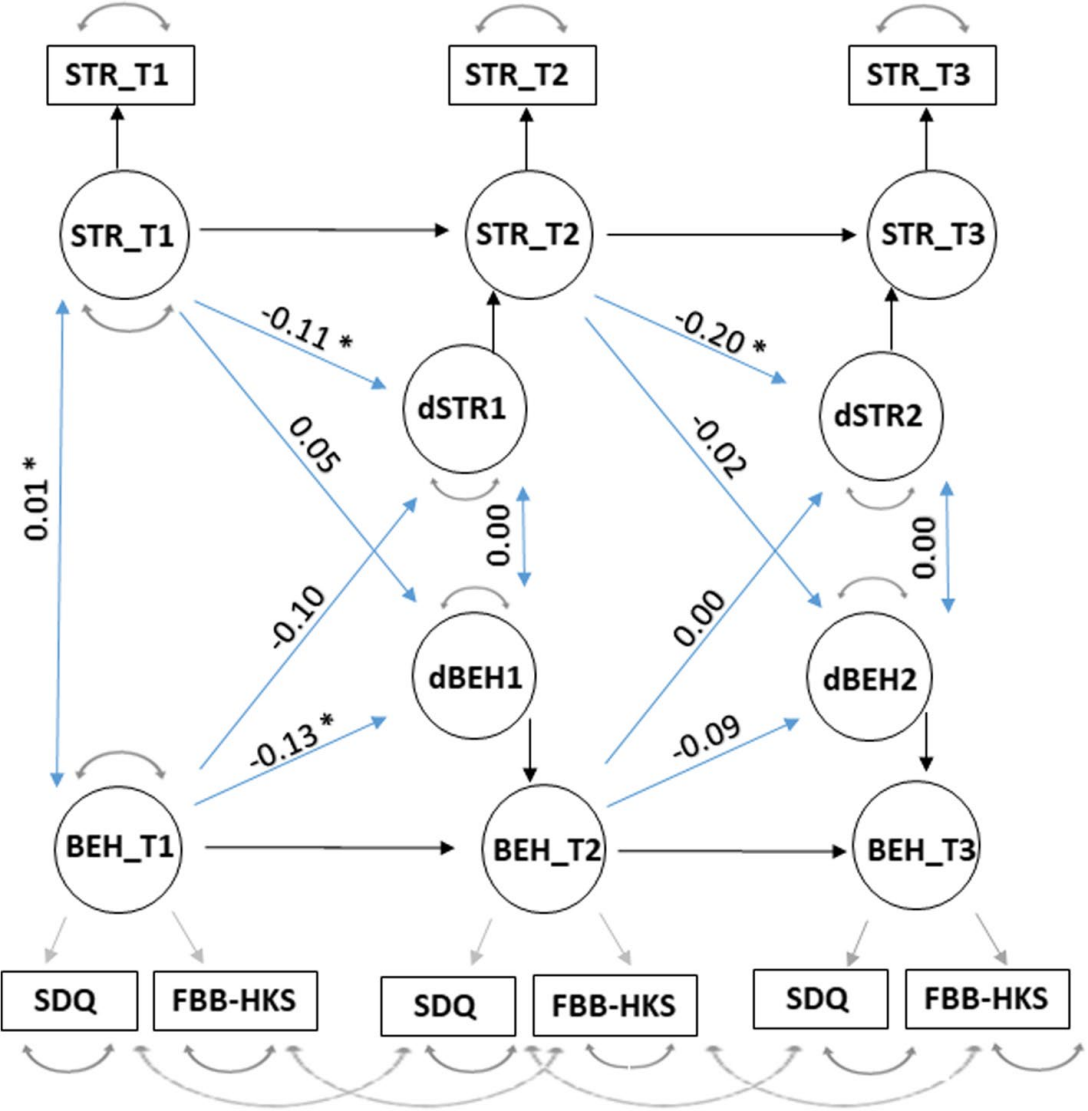
Graphical representation of the longitudinal cross-lagged model used in this study. Latent variables are represented in circles, and observed variables in rectangles. One-pointed arrows indicate directed relationships (factor loadings, regressions) and two-pointed arrows undirected ones (variance, covariance, error). BEH_T1, BEH_T2 and BEH_T3 stand for child behavior problems in waves 1, 2 and 3, respectively (i.e. when children were aged 7, 8 and 10 years). dBEH1 and dBEH2 represent changes of this variable across waves (i.e. from wave 1 to wave 2, and from wave 2 to wave 3, respectively). STR_T1, STR_T2 and STR_T3 stand for maternal stress levels in waves 1, 2 and 3, and dSTR1 and dSTR2 represent changes of this variable from wave 1 to wave 2, and from wave 2 to wave 3). SDQ and FBB-HKS stand for the two sets of observed measurements for child behavior problems (i.e. the Strength and Difficulties Questionnaire and the Fremdbeurteilungsbogen für hyperkinetische Störungen questionnaire). Estimates are reported for all the cross-domain parameters (i.e. linking maternal stress levels and child behavior problems) and the autocorrelation parameters (i.e. linking both variables to the same variable in the preceding wave), with asterisks denoting a significant relationship. Other values are omitted for visual clarity (Table S2 in Suppl. Material)

The longitudinal cross-lagged model in Fig. 3 allowed us to assess the dynamic relationship between maternal stress and child behavior problems during development. The model had a good fit (χ^2^ = 44.18, *df* = 24, RMSEA = 0.056, CFI = 0.985, TLI = 0.976, SRMR = 0.032), which was significantly better than the one of the corresponding univariate model (χ^2^ = 20.12, *df* = 11, *p* = 0.044). Variances in the cross-lagged model showed significant inter-individual differences in maternal stress and child behavior in the first wave (both *p* < 0.001), and in their rate of change between wave 1 and 2 (both *p* ≤ 0.002), and between wave 2 and 3 (both *p* ≤ 0.001; Table S2 in Suppl. Material). Intercept values showed significant mean level changes in maternal stress (but not in child behavior), with stress increasing from wave 1 to wave 2 (*p* = 0.013; *est* = 0.05, *se* = 0.02, *z* = 2.48) and from wave 2 to wave 3 (*p* < 0.001; *est* = 0.09, *se* = 0.02, *z* = 4.00; Table S2 in Suppl. Material).

Inspection of the four parameters linking maternal stress and child behavior across waves showed no significant cross-domain relationships (stress to behavior coupling in waves 2 and 3: *p* = 0.093 and *p* = 0.415; behavior to stress coupling in waves 2 and 3: *p* = 0.260 and *p* = 0.969; estimate of correlated change in waves 2 and 3: *p* = 0.115 and *p* = 0.118), except for stress-behavior covariance in the first wave (*p* < 0.001; *est* = 0.01, *se* = 0.00, *z* = 5.98; Fig. 3; Table S2 in Suppl. Material). In other words, higher maternal stress was linked to higher behavior problems in children at 7 years of age, and although maternal stress and child behavior problems were always significantly correlated in the three waves (Spearman exact test: all *p* < 0.001, *N* = 228, with rho ranging from 0.229 to 0.402), these relationships failed to reach significance after taking into account the other dynamic parameters (see non-significant estimates of correlated change in Table S2 in Suppl. Material). Furthermore, auto-regression parameters for both maternal stress and child behavior showed significant autocorrelation of stress and behavior. In particular, changes in maternal stress between waves 1 and 2 depended on maternal stress in the first wave (*p* = 0.044; *est* = -0.11, *se* = 0.05, *z* = -2.01), and changes in maternal stress between 2 and 3 depended on maternal stress in the second wave (*p* < 0.001; *est* = -0.20, *se* = 0.05, *z* = -3.83; Fig. 3; Table S2 in Suppl. Material). Moreover, changes in child behavior between waves 1 and 2 depended on child behavior in the first wave (*p* = 0.033; *est* = -0.13, *se* = 0.06, z = -2.13), although this relationship was not significant between waves 2 and 3 (*p* = 0.231; Fig. 3; Table S2 in Suppl. Material). Finally, we tested whether these relationships between maternal stress and child behavior differed for children with or without older siblings by using a multi-group model. Although the multi-group model had a good fit (χ^2^ = 66.23, *df* = 49, RMSEA = 0.049, CFI = 0.988, TLI = 0.985, SRMR = 0.036), including the effect of siblings did not provide a significant increase of fit as compared to the less complex cross-lagged model from Fig. 3 (χ^2^ = 19.41, *df* = 25, *p* = 0.777). Therefore, we retained the latter model and refrained from further comparisons between children with and without older siblings.

## Discussion

In this study, we used longitudinal data from 373 mother–child pairs to investigate the complex link between socio-environmental stressors, maternal stress and child behavior problems, in order to detect potential risk factors and protective or promotive factors. First, we assessed whether maternal satisfaction with the environment they lived in (in terms of living standards and quality of social relationships with neighbors) predicted maternal stress levels. Second, we tested whether prenatal stress predicted the occurrence of behavior problems during childhood, and how maternal stress and child behavior problems dynamically affected each other during development. Finally, we assessed the role of siblings as a protective factor buffering the negative effects of maternal stress on child behavior, or generally promoting a healthy child development.

Partially in line with our predictions, mothers reported higher levels of perceived stress when they were not satisfied with the quality of the environment they lived in (Model 1). In particular, the lack of sufficient social areas in the neighborhood (including playgrounds, meeting venues for young people and cultural activities) significantly predicted higher levels of maternal stress. In contrast, other potential stressors (e.g. poor quality relationship with neighbors, low quality of the natural environment, low safety, lack of infrastructures) did not significantly predict maternal stress levels in our study. Incidentally, having a higher number of children also did not predict higher maternal stress levels (Table 1). Previous research has shown a consistent link between environmental quality (e.g. air pollution, lack of green spaces) and individual stress levels [119, 120]. Access to green areas, for instance, is known to buffer the relation between income-related deprivation and mortality in other countries [120]. In our study, however, it was the presence of social meeting places and cultural offers in the neighborhood (rather than the quality of the natural environment) that mostly exerted a positive impact on maternal stress levels. Our study was conducted in Leipzig, which is a relatively small city (about 600,000 inhabitants) with a high proportion of greenness (i.e. on average, 16 m^2^ green space per inhabitant [121]. Therefore, it is possible that the quality of the natural environment is generally high compared to other areas, and the relative importance of social areas on maternal stress may be more important.

In line with our predictions, higher levels of maternal stress were linked to an increased probability of behavior problems in children. In particular, prenatal maternal stress predicted the occurrence of behavior problems during childhood, as measured with both the SDQ and the FBB-HKS questionnaires (Models 2 and 3). These results confirm the long-term negative effect of prenatal stress on child behavior [53, 54, 58–60], which can happen through a variety of mechanisms, from placental signaling to changes in inflammatory and epigenetic pathways (for a review, see [122]). Our results held true in all three waves (i.e. when children were 7, 8 and 10 years), and thus confirm that the negative effects of prenatal stress might last at least into late childhood [56, 58]. This is consistent with previous studies suggesting that prenatal stress, through epigenetic mechanisms or stress hormones directly affecting the placenta and the fetus [45, 46] might have a uniquely powerful impact on child development. Therefore, our study confirms the importance of prevention of prenatal stress and early intervention to improve maternal health and well-being already during pregnancy, in order to reduce the risks of maternal stress and its negative consequences on child development.

In our study, the use of the SDQ and FBB-HKS questionnaires led to similar results, in that prenatal stress predicted the occurrence of behavior problems during childhood when using both the SDQ questionnaire (Model 2) and the FBB-HKS questionnaire (Model 3). These two questionnaires partly measure different behavior problems. In particular, while the SDQ questionnaire assesses both internalizing and externalizing problems (i.e. including behavior problems that span from hyperactivity/lack of attention to emotional symptoms, conduct problems and peer relationship problems [99]), the FBB-HKS questionnaire is more strictly focused on the occurrence of externalizing problems like child hyperkinetic disorders [100]. Therefore, these results suggest that prenatal stress may have a negative impact on a wide range of behavior problems, from hyperkinetic disorders to emotional symptoms, conduct problems and peer relationship problems. In the future, it would be interesting to further investigate whether maternal stress is linked to specific domains of the SDQ questionnaire (e.g. emotional symptoms rather than conduct problems), as internal consistency in this study suggest important variation across these domains.

Also in line with our predictions, higher maternal stress in the tree waves predicted a higher probability of reporting behavior problems in children, as assessed by both the SDQ (Model 2) and the FBB-HKS questionnaires (Model 3). To disentangle whether high maternal stress levels triggered the onset of child behavior problems, or the other way round, or if other external factors explained the link between child behavior and maternal stress, we used longitudinal models. Previous studies have suggested that contingent parental stress may increase the risk that children develop behavioral and cognitive problems [34–36]. In turn, the occurrence of behavior problems in children would increase parental stress [23, 123], triggering a cycle of negative parent–child interactions [8, 34, 76, 77]. Our longitudinal models, however, did not replicate these findings and showed no clear evidence of cross-domain-relationships, except for a significant covariance of maternal stress and child behavior in the first wave. Given that both measures of maternal stress and child behavior problems were based on maternal reports (rather than more objective assessments), caution is needed even when interpreting this cross-domain relationship (e.g. being more stressed, mothers might have wrongly perceived their children as showing more behavior problems). In contrast, we found an interesting autocorrelation effect for both maternal stress and child behavior, with higher maternal stress levels predicting a *decrease* in maternal stress in the following wave, and more behavior problems in the first wave also predicting a *decrease* in behavior problems in the second wave. These results suggest a self-containment effect of stress and behavior problems, with a tendency to return to average levels, perhaps because parents may search for help or take more effective measures when reaching critical stress levels or when detecting high behavior problems in their children.

In line with our predictions on the promotive role of siblings as generally promoting a healthy child development, mothers were overall more likely to report child behavior problems in the absence of older brothers and sisters, independently of their stress levels and child’s gender. Therefore, our results suggest that the presence of older siblings *overall* reduced the likelihood that younger siblings developed behavior problems during childhood, rather than acting as social buffers in case of maternal stress (i.e. the presence of older siblings had a significant main effect in Model 3). This effect was however limited to Model 3, in which child behavior problems were assessed through the FBB-HKS questionnaire (i.e. focusing on externalizing problems such as hyperkinetic disorders [100]), and did not extend to Model 2, in which child behavior problems were instead assessed through the SDQ questionnaire (i.e. including both internalizing and externalizing problems [99]). Therefore, it is possible that the promotive function of older siblings may be limited to externalizing problems.

Interestingly, the presence of older siblings was a significant negative predictor of child behavior problems in Model 3, but the interaction between contingent maternal stress levels and presence of older siblings was not significant, neither in Model 2 nor in Model 3. Therefore, the presence of older siblings overall reduced the likelihood that children developed behavior problems during childhood, but did not specifically mitigate maternal stress. Hence, we agree with authors suggesting an important promotive function of older siblings (see [13, 14, 87]). These results are confirmed by the lack of a significant increase of fit when using multi-group models (see the non-significant comparison between the multi-group and the cross-lagged models). This suggests that the presence of older siblings, while being generally linked to a lower occurrence of child behavior problems (Model 3), failed to modulate the negative effects of maternal stress (as instead found by Gass and colleagues [15]). The direct link between presence of older siblings and lower incidence of child behavior problems may be easily explained with existing literature. By interacting with their older siblings, children may develop better emotional, perspective taking and problem solving skills [123, 124], which are linked to higher social competence and emotion understanding also later in life [125, 126]. Moreover, it is possible that the presence of older siblings provided learning opportunities for parents, affecting their expectations and behavior toward younger offspring (see [14]). Parents with previous experience with adolescent offspring, for instance, are less likely to expect behavior problems during adolescence in younger children [127]. Therefore, a lower incidence of behavior problems in children with older siblings may also depend on different expectations and better parental skills [128].

Finally, mothers were more likely to report child behavior problems for male offspring, with both questionnaires (Models 2 and 3). Our results are not surprising, as sons have often been reported to show more behavior problems than daughters, especially in terms of hyperactivity and lack of attention [101, 129]. In our opinion, however, these results should warn us against the risk that current questionnaires on child behavior might inappropriately categorize male behavior as being problematic (or even suggestive of serious behavioral disorders), simply because the needs of children (both boys and girls) are unlikely to be satisfied in modern Western cultures.

The main strength of our study is the availability and use of longitudinal data from mother–child pairs with several follow-ups from birth until the age of 10. However, in the future, it would be interesting to include biological measures of individual stress rather than self-reported ones and better differentiate between different forms of stress (e.g. acute, episodic, chronic), in order to specifically investigate the promotive and/or protective function of siblings and other members of the social network during child development. Moreover, it would be important to better understand how the role of siblings and other family members differs in different cultural contexts, as there is substantial variation in the way sibling relationships are valued across cultures [130]. In this regard, it would be especially interesting to include measures that specifically assess the quality of sibling relationships, while still using a longitudinal approach, as this allows to better disentangle the complex interplay of parental stress, child behavior problems and several other factors through time. Furthermore, our study sample was unfortunately limited, in that it only included mother–child pairs living in Germany. However, there may be important cross-cultural differences in the link between maternal stress, the occurrence of child behavior problems and the promotive role of older siblings. The promotive function of older siblings, for instance, may be even stronger in cultures where siblings spend more time with each other, or in populations at increased risk of negative stress responses, like families with lower family income or education level [102, 103]. Finally, considering that small household sizes with only one child have become increasingly common in several countries (e.g. in Europe, North-America, China [131]), future studies should particularly focus on how the lack of siblings may affect child development and, especially, on the identification of other promotive factors that may avoid the emergence of internalizing and externalizing problems in children, even in the absence of siblings.

Overall, our study largely confirms previous research on the relationship between social and environmental stressors, maternal stress levels and child behavior, and thus contributes in highlighting the importance of long-term policies of prevention of prenatal stress and early intervention, aimed to increase maternal well-being and reduce the risks of maternal stress already during pregnancy. In particular, this study emphasizes the important role that siblings may have in the family to increase the likelihood of a healthy development for children, and calls for public health policies that directly target children and their siblings, and aim to promote their general well-being and a healthy environment for the development of high-quality sibling relationships.

## Supplementary Information

**Additional file 1:** **Table S1**. Main characteristics of the LINA sub-cohort analyzed in this study, overall and separately for each wave. **Table S2**. Results of the longitudinal model, including estimates, standard errors (SE), *z* values and *p* values. Significant test predictors are marked with an asterisk and are in bold in the *p *column. **Figure S1**. Maternal stress at testing time as measured with the PSQ questionnaire, as a function of parental satisfaction about the social areas in the enviroment they live in (from 0 to 1, i.e. from low to high satisfaction). Circles represent individual responses, and the dashed black line the fitted model unconditional on all the other predictors. Data were jittered horizontally to avoid overplotting. **Figure S2**. (a) SDQ child behavioral index (from 0 to 1, i.e. from less to more problematic), as a function of prenatal maternal stress, and (b) as a function of maternal stress at testing time; and (c) FBB-HKS child behavioral index (from 0 to 1, i.e. from less to more problematic), as a function of prenatal maternal stress, and (d) as a function of maternal stress at testing time. In all cases, maternal stress was measured with the PSQ questionnaire. Individual responses are represented with black circles for mothers of female children, and with grey crosses for mothers of male children. The dashed black line represents the fitted model unconditional on all the other predictors. Data were jittered horizontally to avoid overplotting.

## Data Availability

The datasets used and/or analyzed during the current study are available from the corresponding author on reasonable request.
