# Supplementary material for: Maternal stress, child behavior and the promotive role of older siblings
Source: BMC Public Health. 2022 Apr 29;22:863. doi: 10.1186/s12889-022-13261-2 (PMC9055772; doi:10.1186/s12889-022-13261-2)

**Table S1**. Main characteristics of the LINA sub-cohort analyzed in this study, overall and separately for each wave.

| **Parameters** |  | **1^st^ wave**  **(age 7)** | **2^nd^ wave**  **(age 8)** | **3^rd^ wave**  **(age 10)** | **Overall** |
| --- | --- | --- | --- | --- | --- |
|  |  | N=372 | N=334 | N=268 | N=373 |
| Sex of the child | Females # | 187 | 165 | 132 | 188 |
|  | Males # | 185 | 169 | 136 | 185 |
| Number of siblings | Total * | 0.80 ± 0.75 | 0.79 ± 0.76 | 0.82 ± 0.74 | 0.80 ± 0.75 |
|  | Older sisters * | 0.16 ± 0.37 | 0.14 ± 0.35 | 0.16 ± 0.37 | 0.15 ± 0.36 |
|  | Older brothers * | 0.15 ± 0.36 | 0.16 ± 0.36 | 0.15 ± 0.36 | 0.15 ± 0.36 |
| Child behavioral problems | SDQ * º | 0.18 ± 0.11 | 0.18 ± 0.11 | 0.18 ± 0.13 | 0.18 ± 0.12 |
|  | FBB-HKS * º | 0.17 ± 0.14 | 0.18 ± 0.14 | 0.17 ± 0.14 | 0.18 ± 0.14 |
| Maternal stress | PSQ: prenatal * º | 0.31 ± 0.16 | 0.31 ± 0.16 | 0.31 ± 0.16 | 0.31 ± 0.16 |
|  | PSQ: in the three waves * º | 0.43 ± 0.19 | 0.42 ± 0.19 | 0.41 ± 0.18 | 0.42 ± 0.19 |
| Satisfaction with social and environmental factors | Relationship with neighbors * º | - | - | 0.64 ± 0.17 | - |
|  | Natural environment * º | - | - | 0.73 ± 0.19 | - |
|  | Safety of the area * º | - | - | 0.74 ± 0.20 | - |
|  | Availability of social areas * º | - | - | 0.60 ± 0.20 | - |
|  | Availability of shops * º | - | - | 0.71 ± 0.21 | - |
|  | Availability of other infrastructures * º | - | - | 0.71 ± 0.16 | - |
| Monthly income | Low # | - | - | 24 | - |
|  | Intermediate # | - | - | 88 | - |
|  | High # | - | - | 135 | - |
| Maternal education level | Primary education | 1 | 1 | 1 | 1 |
|  | Lower secondary education | 4 | 4 | 3 | 4 |
|  | Upper secondary education | 131 | 116 | 93 | 132 |
|  | Post-secondary non-tertiary education | 80 | 75 | 62 | 80 |
|  | Tertiary education (1st stage) | 152 | 136 | 107 | 152 |
|  | Tertiary education (2nd stage) | 4 | 2 | 2 | 4 |

The numbers included in the Overall column include all participants who participated in at least one of the three waves, so that information was available on both child behavior and maternal stress levels. Monthly income (i.e. household income per month) was categorized as "low" (<2000 €), "intermediate" (2000-3500 €) or "high" (>3500 €). # stands for number of participants, * stands for mean ± SD, and º stands for indices ranging from 0 to 1.

**Table S2**. Results of the longitudinal model, including estimates, standard errors (SE), *z* values and *p* values. Significant test predictors are marked with an asterisk and are in bold in the *p* column.

| **PARAMETERS** | **Estimate** | **SE** | ***z* values** | ***p*** |
| --- | --- | --- | --- | --- |
| **Variances** | | | | |
| BEH_T1 | 0.01 | 0.00 | 7.82 | **<0.001*** |
| dBEH1 | 0.00 | 0.00 | 4.27 | **<0.001*** |
| dBEH2 | 0.00 | 0.00 | 3.48 | **0.001*** |
| STRl_T1 | 0.03 | 0.00 | 11.70 | **<0.001*** |
| dSTR1 | 0.01 | 0.00 | 3.10 | **0.002*** |
| dSTR2 | 0.01 | 0.00 | 4.03 | **<0.001*** |
| **Intercepts** | | | | |
| BEH_T1 | 0.18 | 0.01 | 32.95 | **<0.001*** |
| dBEH1 | 0.00 | 0.01 | 0.26 | 0.795 |
| dBEH2 | 0.02 | 0.01 | 1.80 | 0.071 |
| STRl_T1 | 0.43 | 0.01 | 43.87 | **<0.001*** |
| dSTR1 | 0.05 | 0.02 | 2.48 | **0.013*** |
| dSTR2 | 0.09 | 0.02 | 4.00 | **<0.001*** |
| **Cross-domain relationships** | | | | |
| dBEH1 ~ STR_T1 | 0.05 | 0.03 | 1.68 | 0.093 |
| dBEH2 ~ STR_T2 | -0.02 | 0.03 | -0.82 | 0.415 |
| dSTR1 ~ BEH_T1 | -0.10 | 0.08 | -1.13 | 0.260 |
| dSTR2 ~ BEH_T2 | 0.00 | 0.09 | -0.04 | 0.969 |
| BEH_T1 ~~ STR_T1 | 0.01 | 0.00 | 5.98 | **<0.001*** |
| dBEH1 ~~ dSTR1 | 0.00 | 0.00 | 1.58 | 0.115 |
| dBEH2 ~~ dSTR2 | 0.00 | 0.00 | 1.57 | 0.118 |
| **Auto-regression** | | | | |
| dBEH1 ~ BEH_T1 | -0.13 | 0.06 | -2.13 | **0.033*** |
| dBEH2 ~ BEH _T2 | -0.09 | 0.07 | -1.20 | 0.231 |
| dSTR1 ~ STR_T1 | -0.11 | 0.05 | -2.01 | **0.044*** |
| dSTR2 ~ STR_T2 | -0.20 | 0.05 | -3.83 | **<0.001*** |

BEH_T1 and BEH_T2 stand for child behavioral problems in wave 1 and 2, respectively. dBEH1 and dBEH2 represent changes of this variable across waves (i.e. from wave 1 to wave 2, and from wave 2 to wave 3, respectively). STR_T1 and STR_T2 stand for maternal stress levels in wave 1 and 2, and dSTR1 and dSTR2 represent changes of this variable across waves (i.e. from wave 1 to wave 2, and from wave 2 to wave 3).

**Figure S1**. Maternal stress at testing time as measured with the PSQ questionnaire, as a function of parental satisfaction about the social areas in the enviroment they live in (from 0 to 1, i.e. from low to high satisfaction). Circles represent individual responses, and the dashed black line the fitted model unconditional on all the other predictors. Data were jittered horizontally to avoid overplotting.


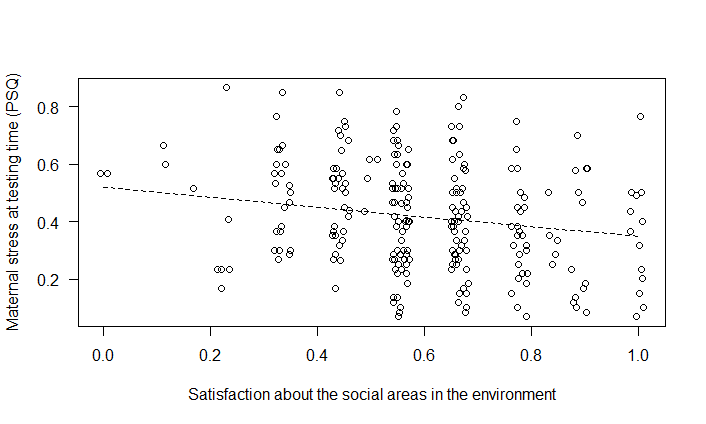


**Figure S2**. (a) SDQ child behavioral index (from 0 to 1, i.e. from less to more problematic), as a function of prenatal maternal stress, and (b) as a function of maternal stress at testing time; and (c) FBB-HKS child behavioral index (from 0 to 1, i.e. from less to more problematic), as a function of prenatal maternal stress, and (d) as a function of maternal stress at testing time. In all cases, maternal stress was measured with the PSQ questionnaire. Individual responses are represented with black circles for mothers of female children, and with grey crosses for mothers of male children. The dashed black line represents the fitted model unconditional on all the other predictors. Data were jittered horizontally to avoid overplotting.


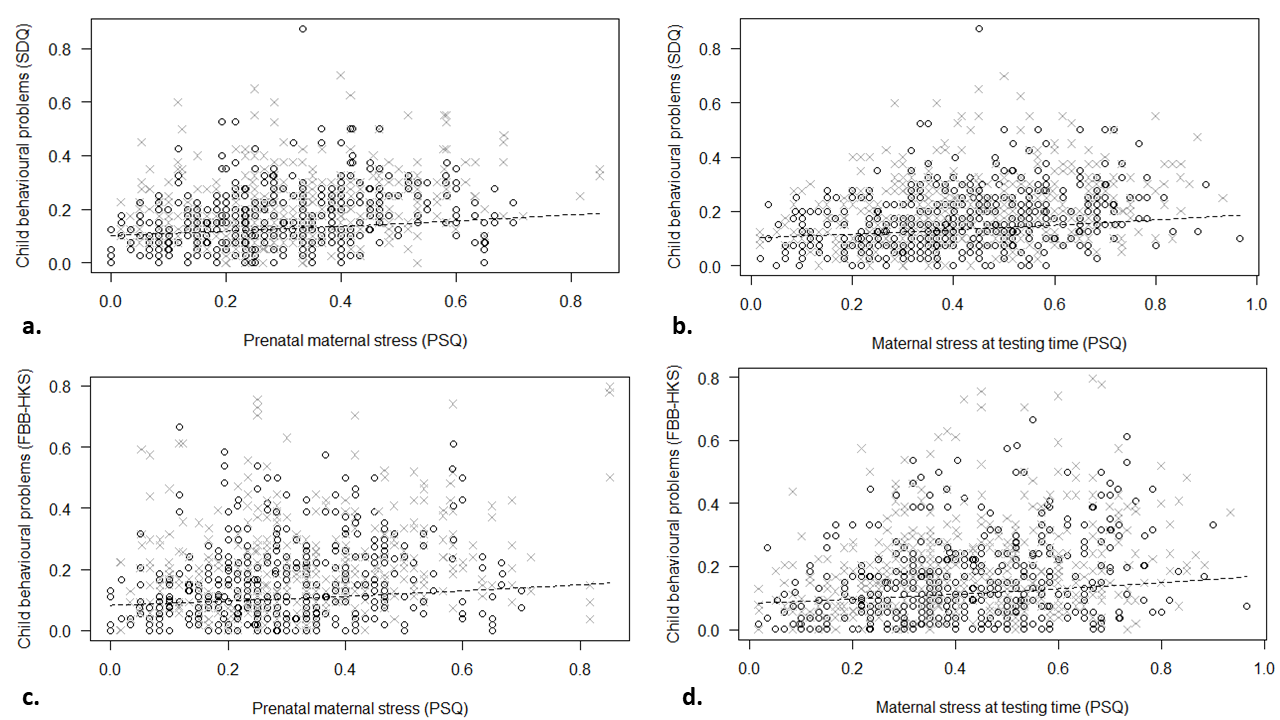

Supplement: Supplementary file 1 — Additional file 1: Table S1. Main characteristics of the LINA sub-cohort analyzed in this study, overall and separately for each wave. Table S2. Results of the longitudinal model, including estimates, standard errors (SE), z values and p values. Significant test predictors are marked with an asterisk and are in bold in the p column. Figure S1. Maternal stress at testing time as measured with the PSQ questionnaire, as a function of parental satisfaction about the social areas in the enviroment they live in (from 0 to 1, i.e. from low to high satisfaction). Circles represent individual responses, and the dashed black line the fitted model unconditional on all the other predictors. Data were jittered horizontally to avoid overplotting. Figure S2. (a) SDQ child behavioral index (from 0 to 1, i.e. from less to more problematic), as a function of prenatal maternal stress, and (b) as a function of maternal stress at testing time; and (c) FBB-HKS child behavioral index (from 0 to 1, i.e. from less to more problematic), as a function of prenatal maternal stress, and (d) as a function of maternal stress at testing time. In all cases, maternal stress was measured with the PSQ questionnaire. Individual responses are represented with black circles for mothers of female children, and with grey crosses for mothers of male children. The dashed black line represents the fitted model unconditional on all the other predictors. Data were jittered horizontally to avoid overplotting. [file 12889_2022_13261_MOESM1_ESM.docx]
